# Dissertation on the Pursuit of Professional Eminence

**Published:** 1841-09

**Authors:** Solyman Brown


### ARTICLE IV.

*Dissertation on the Pursuit of Professional Eminence.* Read before the American Society of Dental Surgeons, at their annual meeting, in the city of Philadelphia, August 10, 1841. By SOLYMAN BROWN, M. D., D. D. S.

THE popular philosophy of mankind, has long assented to the childish assumption, that the *grades* in society, the *diversities* of human attainments, and the *eminence* to which some men arrive in comparison with others, are the arbitrary allotments of nature, or the capricious contingencies of chance.

This doctrine—one of the miserable bantlings of that hoodwinked fatalism which refuses to acknowledge the indissoluble connection between cause and effect, is equally well designed to excuse the folly of that portion of mankind who wander from the pathway of happiness and honour; and to mar the laurels of that other class of men who rise, by their industry, their perseverance and their enthusiasm, to the mountain summit of earthly renown.

The humble soldier bearing his musket among the rank and file of the armies of revolutionary France, and acting as a mere piece of machinery, had no better conception of the agencies which transferred the pupil of the polytechnic school to the throne of the empire, than that the "Little Corporal," was the soldier of fortune. He was not capable of tracing the successive steps by which this consummate commander contrived to elevate himself, with an industry that knew no fatigue, and a zeal that recoiled at no disappointment, above all the experienced generals and venerable marshals of his adopted country, who unanimously conspired, with shouts of acclamation, which caused Europe to tremble from the Baltic to the Euxine, to reward his *republican* virtues with an imperial crown.

Bonaparte, like all other men, was the artificer of his own fortunes, under the uniform law of superintending Providence, that, in like circumstances, like causes produce like effects.

If he became a general in Italy, it was because he had thundered at Toulon with a voice that awoke a sleeping nation to the magnitude of its destinies. If he conquered at Lodi, it was because he led his troops over its memorable bridge into the cannon's mouth. If he obtained the hand of the archduchess of Austria, together with a dispensation from the pope to sanctify his bigamy, it was because he had humbled both her father and his Holiness of Rome in the dust of his fields of battle.

On the other hand—if the star of his fortune went down in eternal night on the plains of Moscow and Waterloo, and if he perished miserably on a barren rock of the ocean, it was because he had outraged every form of religion and of law to gratify an insatiable ambition; repudiated and divorced the amiable Josephine without a crime; and, in the name of liberty, constituted himself a tyrant.

Cause and effect are just as manifest in all the history of the aspiring Corsican, as in the germinations, growth and fructification of the simplest plant, the seed of which is cast into the earth, and the perfection of which is ultimated by the elements. Nor is the instance of Napoleon by any means an exception to any general rule; the fortunes of the most insignificant individual of the human race, are regulated by the same great law of nature.

For the sake of making myself distinctly understood, I will advert to another example: for, as the inimitable Laurence Sterne, has justly remarked—"although an illustration is not a demonstration, nor the wiping of a looking-glass, a syllogism, yet both of them enable us to see more clearly."

Passing over the instance of Columbus, who became the discoverer of the new world because he was the most accomplished and daring navigator of his age, impelled by a perseverance of purpose which no discouragement could countervail; I advert to a more recent example and one not less in point.

Robert Fulton, effected a solution of the very difficult problem of applying the power of steam to the propulsion of boats, as a resulting and incidental effect of his protracted and indefatigable endeavours to construct torpedoes, and move them through the water, as well beneath as upon its surface, for the dispersion and destruction of hostile fleets.

Mechanics and aquatic machinery had been long the absorbing subjects of his reflections and experiments, and the transition was extremely natural from the explosion of his favourite system of sub-marine warfare, to the subject of his great invention.

But the mere mechanical preparation which he brought to bear upon his arduous undertaking, would have been of no avail, if he had not possessed that indomitable perseverance which could sustain discouragement, opposition, and ridicule, without dismay. When his individual resources were exhausted by tedious and expensive experiments, he had the talent to call to his aid the co-operation of the rich and the powerful. When the resources of his own country were inadequate to his purpose, he sought a foreign land; and when one of the great maritime nations of Europe refused or neglected to second his exertions, he repaired confidently to another.

If we trace with care the successive steps by which he arrived at the goal on which his imagination had so long rested with hope, we shall not fail to discover that no other individual of whom the history of the past gives us any account, pursued so firmly and incessantly the only practicable path to the particular tablet on the pyramid of fame, upon which he has inscribed his autograph.

Robert Fulton succeeded in bringing into general use the power of steam as applied to boats, because he employed more skilfully than any other individual who had prosecuted the same design, the means proper to that result. Why is John Milton the greatest poet who has employed the English tongue? Because by his industry and research he made himself decidedly the most learned man that ever courted the British muse. Why did Cicero stand pre-eminent among the orators of the Julian and Augustan ages? Read his luminous and voluminous productions on every subject connected with his high vocation, and you will learn the cause.

Depend upon it, my brethren, that in spite of all the contingencies of chance, and all the caprices of fortune, cause and effect have always existed, in most intimate connection, in all the history of man, and in all the providence of God.

There are, indeed, and always have been, fabulous legends in the realms of romance, concerning entities, without origin, and things created out of nothing; but I may safely challenge authentic history in any department of nature, or of art, to substantiate a single example, not merely of an effect without a cause, but of an effect without its adequate and appropriate cause.

In applying the foregoing principles to the subject of these remarks, which is the attainment of professional eminence, I shall be studiously brief, inasmuch as the time of the members of this society is quite too valuable to be wasted on empty declamation, or wire-drawn argument. Yet, I indulge the hope that a few moments assigned by our by-laws to this object, may be profitably spent in considering the means by which certain individuals in every department of human enterprise, and in our own profession as well as in others, obtain distinction and pre-eminence above their fellows.

The objects or ends for which this pre-eminence is sought, are various:—some benevolent, others selfish:—and these ends are, in all cases, wholly distinct from the means by which they are attained.

I wish it, therefore, to be distinctly understood, that, while I speak of professional eminence, and the means necessary to be employed, I neither justify, nor condemn the object of pursuit; because the complexion of this, depends wholly on the moral character of the motives.

If an individual aim at professional distinction for the sake of the pre-eminent uses which distinction will enable him to perform, his motives are virtuous in an eminent degree:—whereas, if personal aggrandizement, and the desire to rise at the expense of others' welfare, be the moving impulses of the mind, the morality of the motive is of a very questionable character.

It is for this reason that I shall limit my remarks, on this occasion, to the means of professional eminence, without regard to its end.

Again:—I shall not take it upon me to decide, on this occasion, whether permanent and real prosperity pertains most truly to exalted pre-eminence in professional notoriety and success, or to that happy mean which is equally removed from dangerous extremes.

Neither do I undertake to determine whether the prosecution of any one exclusive object of human pursuit, to the neglect of many other important ones, is conducive to the enlarged and permanent happiness of man.

My object shall be, simply, to trace the steps by which the giddy summit of renown, may be the most certainly attained.

And, in the first place, the individual who wishes to run, successfully, this race of aspiring ambition, must be careful in the outset to select the calling, for which early impressions and subsequent education shall have best prepared him. There is quite as much truth as poetry in the well-known hemistich:—

"Just as the twig is bent, the tree 's inclined," Bonaparte never could have become the founder of the respectable religious sect, known as Methodists; nor could John Wesley have consolidated a mighty empire on the ruins of the French Revolution, unless the early impressions, habits, and education of both, had been reversed, so that Wesley should have issued from the Polytechnic school, and Bonaparte, from the University of Oxford.

The early habits of Washington but illy prepared him to become the rival of Fulton or Whitney, in the successful combination of useful machinery ; nor were either of the latter at all prepared by primary inculcations and habits of mind, to unfurl the banner of universal freedom on the castellated battlements of the world.

On this principle it is that I urge the necessity of making a suitable selection from among the numerous avocations of civilized society, in order to secure the first necessary step in the road to distinction. A mistake in this point, will of necessity be fatal. If Newton and Shakspeare had exchanged profession both would have remained alike unknown to fame. Subsequent exertions have little influence in correcting a fundamental error. The superstructure is nothing better than a house built upon the sand. There can be no reasonable question that in this, as in ordinary wedlock, more than one half at least of the professional abortions in civilized society, result from incongruous unions.

Else, why should a Cæsar Borgia, ever become a prelate ; or a Richard Cromwell, lord protector of England? On what other principle did an imbecile Xerxes bring the myriads of Asia to fatten the fields of Greece, or the stupid cabinet of George the III. of England, attempt to subjugate the freemen of America? There may have been men in Persia, in the days of that unfortunate monarch, who could have led those countless armies to certain victory; as we know there were statesmen in England in the days of Lord North, who could have imparted to the ear of majesty, more salutary counsels. Let therefore, every individual who indulges the ambition of rising to professional distinction, see well to it, that he is not a Mercury attempting to handle the thunderbolts of Jupiter; for, although the former was one of the accredited gods of Olympus when he burned his fingers, he was meddling imprudently with the attributes of another.

In the second place: If any man would aspire to professional pre-eminence, he must have a singleness of purpose which concentrates all his energies to the object of pursuit, to the total or partial exclusion of every thing besides. The true path having once been found, it must be steadily pursued in preference to all the cross-roads and by-ways that may divert for a moment the attention of the traveller. It may, indeed, be long and tedious. Its very directness may induce fatigue. It may be barren of wayside verdure, over-arching shades, and exhilirating flowers. The lateral paths, on the contrary, may abound with tempting objects, enticing the beholder to turn aside for a moment in order to revive the senses with fairer scenery and fresher gales. But all these seductive allurements must be repulsed with a stoical indifference, and a steady eye must be kept upon the shining goal at the end of the race.

As well might the general of an army attempt to disperse the Grecian phalanx by sending his troops in scattered confusion over the field of battle, as the professional aspirant, presume to over-match his competitors by dividing his energies among a diversity of pursuits.

Alexander was a soldier, Homer a poet, Mozart a musician, and Newton a mathematician, and neither of them either was, or desired to be any thing else. Other men have, indeed, been good generals, good poets, good musicians, and good geometers, without making these pursuits exclusive objects of affection; but the distinction of super-eminent belongs to those who have looked with a steady eye at a single object.

It, indeed, seems sometimes difficult to determine which among various objects of ambition, must be accounted the means, and which the end:—as for example, when an individual, high in professional distinction, like Sir Robert Astley Cooper,* combines with reputation great pecuniary emolument. It may, in such a case, be avarice, or it may be ambition, which imparts a healing impulse to the mind. But, as that which is a means, is mainly accessory to the end proposed, this seeming diversity of pursuit is perfectly consistent with absolute unity of design. Thus, Cæsar, when he borrowed forty thousand sesterces of a Roman banker to enable him to prepare for an expedition into Spain, declaring to a friend that he needed that sum to make him worth just nothing at all, desired the money to aid in accomplishing the purposes of his ambition; whereas a Rothschild may covet notoriety merely that his celebrity may advance his fortune.

*Of this distinguished individual, so recently numbered among the dead, his biographer, Sir Benjamin C. Brodie, confidently declares "that he derived from his practice a larger income than was obtained by any other person, either in the medical profession or in any other."

This idea of making everything subservient to the principal object of the soul's idolatry, may be elucidated and confirmed by appealing to the history of any individual in any human calling, who has been absorbed in a single pursuit for a course of years. Observation has supplied us all with numerous instances of ultimate success attendant upon this singleness of design.

It is no part of my present purpose to apply these remarks to any other profession than that in which the members of this society are engaged. Let it suffice, therefore, to say, that the dental practitioner who aims at unrivalled distinction in his art, must "lay aside every weight" that would either retard his progress, or diminish the elevation of his flight. Is he fond of fashionable society—he must sacrifice his pleasure in this behalf and become the steady companion of his instruments, his patients, and his books. Has he any relish for the turmoil of political strife—the haunt of the politician must be exchanged for the laboratory of his art. Does he love hours of listless leisure and dreaming repose—he must shake himself from the cobwebs of drony apathy, and devote at least three-fourths of his hours to business, exercise, and study. Is he fond of the reveries of music and of song—he must hang his harp upon the willows, and abandon melody to the winds. Has he by early habit become enamoured of the long and luscious dinners of the epicure, and of the midnight revels of the bacchanalian—he must learn that lesson so difficult for young adventurers in the career of life, and restrain those wayward appetites which wage eternal warfare with fame and fortune. Is his heart susceptible of friendship—his companions must be those alone who can promote his design of greatness; and should he resolve to engage in the tenderest of mortal or immortal ties, and adopt a conjugal companion, he must seek for one who can suspend necklaces of gold and diamonds upon the idol of his ambition. In short, he must even divorce his Josephine, if a Maria Louisa can better secure the perpetuity of his fame.

Descending from these general principles of action, and fixing his attention on more minute particulars, he must never forget to arouse himself from his pillow at the dawn of day, and attire himself with care as becomes the gentility of his calling; after preparing himself by ablutions like the Roman athlætæ, for the vigorous prosecution of his diurnal course—to be ever on the alert to convert every thing to his use, bend every thing to his purpose, and transmute all the events of each successive day into so many ascending steps in the ladder of his ambition, so that, at the hour of midnight, when he has taken an account of his daily doings, he may throw himself upon his couch in exulting confidence that he has not lost an hour.

In the duties of his profession he must not only perform all parts of his work in the most approved and substantial manner, but he must impress the public with the value and importance of his individual services; invest his simplest operations with an air of mystery, and conceal his infirmities in the mists and combings of that prosperous billow upon which his bark is bearing him to the haven of renown.

Do any of my fraternal auditors whisper in my ear, that on conditions like these they cannot consent to labour their way to the mountain summit of professional pre-eminence? Be it so.

It is not for me to question either the virtue or the prudence of their choice. That idiosyncrasy of mental temperament which thirsts for exaltation in any single department of human knowledge or skill, to the neglect and contempt of every thing besides, may not, peradventure, afford the surest criterion of either wisdom or felicity.

There may be those, even in this little circle, who are not inclined to lavish the mighty energies of an immortal mind in any single merely temporal pursuit. They may deem it proper to make an outlay of just sufficient application and talent to the call- which affords them maintenance; while other objects of at least co-ordinate importance, command a portion of their powers. They may be satisfied with mediocrity, or moderate eminence in their particular profession, on the condition that they superadd to this, other attainments of a valuable character. Some may desire to segregate from professional confinement a few happy hours in each successive day for exercise and recreation, deeming health of body and elasticity of mind, invaluable possessions. They may wish to add to these a few other hours to be consecrated to the claims of friendship, society and religion. Elegant literature may intrude its pretensions to occasional regard. The splendid developments of modern science, and the curious incidents of ancient history may come in, at times, for a share of intellectual devotion. Or, peradventure, the moral and intellectual culture of a rising family; the social and innocent pleasures which may be shared with a lovely wife and amiable children, may sometimes beguile a happy hour from the impious worship of the Moloch of wealth and the Dagon of ambition.

All these, and many more, are the enticing allurements by which an active imagination, and peradventure, the most solid judgment, may well be alienated from the ardent pursuit of that excess of professional celebrity, which a breath of detraction or the touch of death may dissipate forever!

But, although all the wise and good among my professional compeers, may not be inclined to sacrifice every other object of honourable pursuit in order to attain that solitary pre-eminence which rises in envied grandeur above the ordinary summits of human attainment, like an island of ice above the surrounding billows; which the sun, indeed, gilds with splendor, but which mortals avoid in dismay;—yet the noble mind would feel itself debased in any calling, to remain at a great remove from that honourable distinction which industry, talent and virtue may most certainly attain.

Nor is there any danger of instituting too high a standard of professional acquirement, consistent with our other duties to ourselves, to society, and to God; especially in times like these, when a mighty nation, comprising 17,000,000 of people, is literally overrun, from the Canadas to the Gulf of Mexico, and from the Atlantic to the Rocky Mountains, by a host of greedy adventurers, well nigh as numerous and as ignorant as the swarms of Egypt that devoured that fertile land!

Well might we be tempted to regret that we, too, had not an overflowing Nile to wash away their foot-prints, and reanimate the verdure which they have already trodden down!

In place of those multifarious armies which are yearly ushered into our profession after a few weeks preparatory qualification, (I might, perhaps, better say disqualification) in the offices of men whose original preparation was of the same character, may we not indulge a hope that the time will come when the standard established by this society, shall receive that support from an intelligent community, which will insure to the profession of dental surgery, in ages to come, men of education, talent, and integrity? If not, farewell forever to the general utility of dental science! And farewell to the enviable fame which might have attended, to he latest times, its well-instructed and honourable professors!

